# *In vitro *antiplasmodial activity of crude extracts of *Tetrapleura tetraptera *and *Copaifera religiosa*

**DOI:** 10.1186/1756-0500-4-506

**Published:** 2011-11-23

**Authors:** Jean Bernard Lekana-Douki, Sandrine Lydie Oyegue Liabagui, Jean Bernard Bongui, Rafika Zatra, Jacques Lebibi, Fousseyni S Toure-Ndouo

**Affiliations:** 1 Unité de Parasitologie Médicale (UPARAM), Centre International de Recherches Médicales de Franceville (CIRMF) B.P. 769 Franceville, Gabon; 2 Unité de Recherches en chimie, Faculté des Sciences, Université des Sciences et Techniques de Masuku, B.P. 943 Franceville, Gabon; 3 Département de Parasitologie-Mycologie Médecine Tropicale, Faculté de Médecine, Université des Sciences de la Santé, B.P. 4009 Libreville, Gabon; 4 Unité de Parasitologie Médicale Centre International de Recherches Médicales de Franceville (CIRMF), Département de Parasitologie-Mycologie Médecine Tropicale, Faculté de Médecine Université des Sciences de la Santé. B.P. 769 Franceville Gabon

**Keywords:** Plant extracts, Fabaceae, antiplasmodial activity, cytotoxicity, *Plasmodium falciparum*

## Abstract

**Background:**

Malaria remains a major public health problem, especially in tropical and subtropical regions because of the emergence and widespread of antimalarial drug resistance. Traditional medicine represents one potential source of new treatments. Here, we investigated the *in vitro *antiplasmodial activity of bark extracts from two *Fabaceae *species *(Tetrapleura tertaptera and Copaifera religiosa*) traditionally used to treat malaria symptoms in Haut-Ogooué province, Gabon.

**Findings:**

The antiplasmodial activity of dichloromethane and methanolic extracts was tested on *P. falciparum *strains FCB (chloroquine-resistant) and 3D7 (chloroquine-sensitive) and on fresh clinical isolates, using the DELI method. Host cell toxicity was analyzed on MRC-5 human diploid embryonic lung cells using the MTT test.

The dichloromethane extracts of the two plants had interesting activity (IC_50 _between 8.5 ± 4.7 and 13.4 ± 3.6 μg/ml). The methanolic extract of *Tetrapleura tetraptera *was less active (IC_50 _around 30 μg/ml) and the methanolic extract of *Copaifera religiosa *was inactive. The selectivity index (toxicity/antiplasmodial activity) of the dichloromethane extract of *Tetrapleura tetraptera *was high (around 7), while the dichloromethane extract of *Copaifera religiosa *had the lowest selectivity (0.6). The mean IC_50 _values for field isolates were less than 1.5 μg/ml for dichloromethane extracts of both plants, while methanolic extracts of *Tetrapleura tetraptera *showed interesting activity (IC_50 _= 13.1 μg/ml). The methanolic extract of *Copaifera religiosa *was also inactive on field isolates.

**Conclusions:**

Dichloromethane extracts of *Tetrapleura tetraptera *and *Copaifera religiosa*, two plants used to treat malaria in Gabon, had interesting antiplasmodial activity *in vitro*. These data provide a scientific rationale for the traditional use of these plants against malaria symptoms. Bioactivity-guided phytochemical analyses are underway to identify the active compounds.

## Findings

Malaria still kills nearly a million people worldwide each year, and most malarial deaths are due to *Plasmodium falciparum *(WHO 2009 http://www.who.int/features/factfiles/malaria/malaria_facts/fr/index.html). A major obstacle to malaria control is the rapid emergence and spread of antimalarial drug resistance, and new antimalarial compounds are urgently needed. Plants have been used medicinally throughout history, and the two best conventional antimalarial drugs, artemisinin and quinine, are both derived from traditional medicines.

Gabon is a country of about 1.7 million people where malaria transmission is hyperendemic and perennial. Forest occupies 80% of the country and represents a potentially rich source of natural therapeutic molecules. Traditional medicine is a significant part of the Gabonese cultural heritage and is still relied on by a large majority of villagers. Medicinal plants used in the north and south of the country have been listed [1,2].

During an ethno-botanical survey in Haut-Ogooué province, south-east Gabon, we identified firstly nine plants with antiplasmodial activity [3]. Furthermore, traditional healers informed us that two Fabaceae species (*Copaifera religiosa *and *Tetrapleura tetraptera*) were used for this purpose. *Tetrapleura tetraptera *and *Copaifera religiosa *are perennial trees widely distributed in Gabon. Their bark is used to treat various diseases in Haut-Ogooué. Several members of the Fabaceae family are reported to have antiplasmodial activity [4-7].

Here we investigated the antiplasmodial activity and cytotoxicity of dichloromethanic and methanolic extracts of *Copaifera religiosa *and *Tetrapleura tetraptera*.

### Plant material and extraction

Plant extracts were prepared by macerating 100 g of dried and powdered bark at room temperature for about 24 h. The material was extracted sequentially with dichloromethane and then methanol. The quantity of solvent used for each extraction was at least 10 times the quantity of plant material. Thus, two extracts were obtained for each plant. The filtrates were evaporated to dryness under reduced pressure with a rotary evaporator (Rotavapor^®^) at 30°C.

### Parasite culture

*P. falciparum *strains 3D7 (chloroquine-sensitive) and FCB (chloroquine-resistant) obtained from MR4^® ^(Malaria Research and Reference Reagent Resource Center) were cultured in standard conditions[8]. For some experiments the parasites were synchronized by repeated 5% sorbitol treatment. The plant extracts were dissolved in DMSO at an initial concentration of 200 mg/ml and serially diluted with culture medium before being added to synchronous parasite cultures. The range of extract concentrations was 1000 μg/ml, 500 μg/ml, 250 μg/ml, 125 μg/ml, 62.5 μg/ml, 31.25 μg/ml, 15.62 μg/ml, 7.81 μg/ml, 3.90 μg/ml, 1.95 μg/ml, 0.98 μg/ml, 0.49 μg/ml, 0.24 μg/ml, 0.12 μg/ml and 0.06 μg/ml. Two hundred (200) microliters of synchronised trophozoite suspension containing different concentrations of the plant extracts (1.5% final hematocrit in RPMI 1640 medium + 0.5% Albumax^®^) were incubated in 96-well flat-bottom plates (NUNC, VW International, Strasbourg, France) at 37°C for 42 h, as previously described [9]. Parasite growth was stopped by freezing at -20°C.

#### Field isolates

Field isolates were collected with the patients' or guardians' informed consent. Ethical clearance and national endorsement were received from the Gabonese Ministry of Health. Three to five milliliters (3-5 ml) of blood was collected in an EDTA tube, and malaria was diagnosed with the Lambaréné blood smear method [10] with a cutoff of 1000 parasites/μL. The sensitivity of field isolates to dihydroartemisinin (DHA) and chloroquine (CQ) was tested.

### Antiplasmodial activity

Antiplasmodial activity was analyzed with the DELI method (double-site enzyme-linked lactate dehydrogenase immunodetection assay) a pLDH measurement with an ELISA method as previously described [11]. Briefly, frozen parasites were thawed at room temperature for 1 hour. Then 100 μL of lysing buffer and an appropriate volume of sample were added to MAb 17E4-precoated wells before incubation with shaking at 37°C for 1 h. The plate was washed five times and 100 μL of biotinylated MAb 19G7 was added to each well at 37°C for 30 min. The plate was washed and 100 μL of peroxidase-labeled streptavidin was added at 37°C for 15 min. The plate was washed and 100 μL of a mixture (v/v) of a peroxidase substrate solution (3,3',5,5'-tetramethylbenzidine and 0.02% H_2_O_2_) (Kirkegaard and Perry, Gaithersburg, MD) was added and incubated in the dark for 15 min at 37°C. The reaction was stopped with sulfuric acid, and absorbance was read with a microplate spectrophotometer (LP400; Bio-Rad, France) at 450 nm with a reference wavelength of 620 nm. All experiments were performed at least in duplicate. The IC_50 _(drug concentration that reduced parasitemia by 50%) was calculated as the mean of 3 independent experiments in different times.

#### Cytotoxicity assay

The cytotoxicity of the extracts was evaluated on MRC-5 human diploid embryonic lung cells, by using the MTT tetrazolium salt (3-(4,5-dimethylthiazol-2-yl)-2,5-diphenyltetrazolium bromide (Sigma^®^, Germany)) colorimetric method based on cleavage of the reagent by mitochondrial dehydrogenase in viable cells [12]. Cytotoxicity was scored as the percentage reduction in absorbance at 570 nm in treated cultures versus control cultures (contained culture medium only). All experiments were performed at least in duplicate. The 50% cytotoxic concentration (CC_50_, the drug concentration that reduced the number of viable cells by 50%) was calculated as the mean of 3 independent experiments in different times.

#### Selectivity index

The selectivity index (SI), corresponding to the ratio between cytotoxic and antiparasitic activities, was calculated as follows:

$$\text{S}{\text{I}}_{Plasmodium}=\text{ (C}{{\text{C}}_{\text{50}\;}}_{Human}\text{)}∕\left(\text{I}{\text{C}}_{50\;Plasmodium}\right).$$

## Results and Discussion

Several members of the Fabaceae family exhibit antiplasmodial activity, both *in vitro *and in animal models, and some compounds with antiplasmodial activity have been isolated from them [4,6,13-21]. Here, we sought a scientific rationale for traditional use of two Fabaceae species (*Copaifera religiosa *and *Tetrapleura tetraptera*, voucher numbers D. N. 584 and J.J. WIE 6388 respectively from the Gabonese national herbarium) traditionally used to treat malarial symptoms. From 100 g of dried and powdered bark, the extraction yields in dichloromethane were 3.75% and 4.58% for *Tetrapleura tetraptera *and *Copaifera religiosa*, respectively. The yields in methanol, a more polar solvent, were 19.33% and 16.85% for *Tetrapleura tetraptera *and *Copaifera religiosa*, respectively.

The antiplasmodial activities and cytotoxicity of the dichloromethane and methanolic extracts were analysed (Table 1).

**Table 1:** In vitro antiplasmodial activity, cytotoxicity, and selectivity index of the plant extracts

Plants species	Voucher number	Extract	Antiplasmodial activity (IC_50_, μg/ml)^a^		Cytotoxicity MRC-5	Selectivity Index^c^	
			FCB	3D7	(CC_50 _μg/ml)^b^	FCB	3D7
***Copaifera religiosa***	D. N. 584	CH_2_Cl_2_	13.4 ± 3.6	8.5 ± 4.7	4.87 ± 0.5	0.37	0.61
		CH_3_OH	500.7 ± 16.4	480.9 ± 34.2	NT	ND	ND
							
***Tetrapleura tetraptera***	J.J. WIE 6388	CH_2_Cl_2_	10.1 ± 3.2	13.0 ± 5.1	79.9 ± 24.3	7.91	6.15
		CH_3_OH	34.6 ± 4.7	29.6 ± 6.9	89.4 ± 10.8	2.58	3.02
Chloroquine			324.8 ± 16.8 nM	7.9 ± 2.3 nM			
							

a: IC_50_: (Inhibition concentration 50%) is the drug concentration that reduced parasitemia by 50%

b: CC_50_: (Cytotoxic concentration drug 50%) is the drug concentration that reduced the number of viable MRC-5 cells by 50%

c: Selectivity index = Ratio CC_50_/IC_50_

NT: Not Tested

ND: Not Determined

Based on WHO guidelines and previous data [22] antiplasmodial activity was classified as follows: high (IC_50 _<5 μg/ml), promising (5<IC_50 _<15 μg/ml), moderate (15<IC_50 _<50 μg/ml) and inactive (IC_50 _>50 μg/ml).

The dichloromethanic extracts of both plants showed promising activity, indicating that they contained the main active compounds.

The dichloromethanic extract of *Tetrapleura tetraptera *had IC_50 _values of 10.1 ± 3.2 μg/ml and 13.0 ± 3.1 μg/ml for strains FCB and 3D7, respectively. Cytotoxicity on MRC-5 human foetal cells was weak (CC_50 _= 79.9 ± 24.3 μg/ml), giving good selectivity indexes (7.91 and 6.15 for strain FCB and 3D7 respectively). The methanolic extract of *Tetrapleura tetraptera *had moderate antiplasmodial activity (IC_50 _34.6 ± 4.7 μg/ml and 29.6 ± 6.9 μg/ml on FCB and 3D7, respectively), but also weak cytotoxicity on MRC-5 cells (CC_50 _89.4 ± 10.8 μg/ml), giving selectivity indexes of 2.58 and 3.02 respectively. Chloroquine, tested as a control, had IC_50 _values of 324.8 ± 16.8 nM for FCB and 7.9 ± 2.3 nM for 3D7.

*Tetrapleura tetraptera *is widely used in Nigeria, and its fruits have been reported to have nutritional, antiparasitic, antidiabetic, analgesic and antiinflammatory properties [23-25]. People living in Haut-Ogooué use a decoction of *Tetrapleura tetraptera *bark to treat tummy-ache and vomiting, as well as fever and headache. It is also used as a deworming and purgative agent at low doses. People also use this plant around food crops to protect them against pests. The selective antiplasmodial activity of *Tetrapleura tetraptera *bark observed here is consistent with the reported antiplasmodial activity of *Tetrapleura tetraptera *fruit in experimental mice [26]. These fruits contain many compounds with antiplasmodial activity, including triterpeniods and flavonoids [27,28]. The bark may contain triterpenes and other compounds with antiplasmodial properties [29]. The exact mechanism of antimalarial action of flavonoids is unclear, but some flavonoids have been shown to inhibit the influx of L-glutamine and myoinositol into *P. falciparum*-infected erythrocytes [30]. Other flavonoids such as a flavone glycoside from *Phlomis brunneogaleata *and iridoid from *Scrophularia lepidota *have been reported to inhibit the FabI enzyme of *P. falciparum *[31,32]. Triterpenoids such as Iridal extracted from *Iris germanica L*. are suspected to act against the reinvasion step rather than the maturation step of *P. falciparum*, and has cumulative inhibitory effect on the main metabolic pathways of the parasite [33].

To our knowledge this is the first study of the antiplasmodial activity of *Copaifera religiosa*. The dichloromethanic extract of *Copaifera religiosa *showed promising antiplasmodial activity, with IC_50 _values of 13.4 ± 3.6 μg/ml and 8.5 ± 4.7 μg/ml on strains FCB and 3D7, respectively. However, it was also highly cytotoxic for human foetal MRC-5 cells (4.87 ± 0.5 μg/ml), giving poor selectivity indexes of only 0.37 and 0.61. The methanolic extract of *Copaifera religiosa *had no noteworthy antiplasmodial activity (IC_50 _= 500.7 ± 16.4 μg/ml and 480.9 ± 34.2 μg/ml for strains FCB and 3D7).

People living in Haut-Ogooué use *Copaifera religiosa *mixed with lemon juice. The toxicity of the dichloromethane extract raises questions as to the effects of *Copaifera religiosa *when used to treat malaria attacks. However, Gabonese villagers use an aqueous mix that concentrates more compounds than dichloromethane extracts. Certain compounds present in these mixtures might neutralise toxic components, as might metabolic processes after ingestion. Studies using animal models are needed to determine *in vivo *toxicity. As reflected in its name, *Copaifera religiosa *is used by people in southern Gabon during spiritual ceremonies, as an ingredient of facial make-up. Traditional hunters use it as a lucky charm.

As shown in Table 2 the dichloromethanic extracts were also highly active on field isolates of *P. falciparum*: the median IC_50 _values of *Tetrapleura tetraptera *and *Copaifera religiosa *were 0.9 (0.5-5.0) μg/ml and 1.5 (30.3-0.8) μg/ml, respectively. The methanolic extract of *Tetrapleura tetraptera *also showed promising activity, with a median IC_50 _of 13.1 (75.4-9.3) μg/ml, whereas the methanolic extract of *Copaifera religiosa *was inactive (IC_50 _of 227.8 (20-697.6) μg/ml). Mainly for the most isolates, the activity of the extracts did not correlate with parasite sensitivity to CQ or DHA, suggesting that the mechanisms of action of compounds in the plants extracts are different from those of CQ and DHA. Half (50%) the field isolates were chloroquine-resistant (IC_50 _≥ 100 nM), and one had diminished susceptibility to DHA (IC_50 _= 11.1 nM).

**Table 2:** *In vitro *antiplasmodial activity (IC_50_) of Fabaceae extracts on field isolates of *P. falciparum*

	IC_50 _± SD (μg/mL)				IC_50 _± SD (nM)	
	
Isolates	*Tetrapleura tetraptera *(μg/ml)		*Copaifera religiosa *(μg/ml)		DHA (nM)	CQ (nM)
				
	CH_2_Cl_2_	MeOH	CH_2_Cl_2_	MeOH		
**21431**	0.6 ± 0.7	11.0 ± 0.6	1.1 ± 0.4	200 ± 10.6	0.3 ± 0.1	3.4 ± 2.0
**21439**	0.5 ± 0.2	12.6 ± 0.9	0.9 ± 0.5	75 ± 4.8	0.6 ± 0.2	110.0 ± 7.3
**21489**	0.8 ± 0.3	10.2 ± 2.3	1.6 ± 0.8	20 ± 3.5	4.8 ± 1.0	107.7 ± 2.1
**21542**	1.2 ± 0.5	14.7 ± 1.5	3.6 1.3	25.6 ± 2.1	5.8 ± 0.8	143.0 ± 2.3
**21552**	5.0 ± 1.1	75.4 ± 8.6	30.3 ± 1.3	400 ± 23.4	0.9 ± 0.2	100.0 ± 9.3
**21657**	0.9 ± 0.4	23.8 ± 9.4	9.1 ± 1.6	236 ± 9.7	2.0 ± 0.5	3.6 ± 0.8
**21660**	1.2 ± 0.2	13.5 ± 7.8	0.9 ± 0.3	88 ± 10.2	0.6 ± 0.3	212.8 ± 3.9
**21676**	1.0 ± 0.5	9.3 ± 0.9	2.4 ± 1.7	45.2 ± 1.8	5.9 ± 1.8	183.8 ± 12.8
**21679**	2.1 ± 0.5	11.2 ± 2.6	1.1 ± 0.3	320.7 ± 9.3	6.4 ± 1.1	278.5 ± 5.4
**21681**	0.7 ± 0.6	12.5 ± 3.4	0.9 ± 0.6	264.6 ± 8.4	11.1 ± 1.7	29.5 ± 10.4
**21683**	1.7 ± 0.4	13.8 ± 1.4	1.5 ± 0.2	321.3 ± 3.1	1.0 ± 0.4	70.6 ± 2.6
**21700**	2.6 ± 1.0	14.9 ± 0.7	2.7 ± 0.6	200.1 ± 6.0	1.9 ± 0.4	7.8 ± 2.1
**21706**	0.8 ± 0.3	11.7 ± 1.6	3.6 ± 1.5	697.6 ± 8.2	1.0 ± 0.3	375.2 ± 4.3
**21721**	0.7 ± 0.3	9.9 ± 0.8	1.3 ± 0.5	345.7 ± 13.6	0.1 ± 0.2	60.0 ± 2.3
**21743**	1.8 ± 1.4	14.2 ± 3.7	2.6 ± 0.6	294.3 ± 7.3	0.6 ± 0.2	17.9 ± 1.8
**21782**	0.7 ± 0.3	15.9 ± 0.9	0.8 ± 0.6	219.6 ± 20.3	2.1 ± 0.6	26.6 ± 2.1
Median (Min-Max)*	0.9 (0.5-5.0)	13.1 (9.3-75.4)	1.5 (0.8-30.8)	227.8 (20.0-697.6)	1.45 (0.1-11.1)	85.3 (3.4-375.2)

Median (Min-Max)*: median of IC_50 _with minimum and maximum.

**DHA: **dihydroartemisinin.

**CQ**: chloroquine.

This preliminary analysis of *Copaifera religiosa *and *Tetrapleura tetraptera *shows that both Fabaceae have promising antiplasmodial activity. Although the mechanisms of action of these plants have not yet been identified, some plants are known to exert their antiplasmodial action by increasing red blood cell oxidative stress [34] or by inhibiting protein synthesis [35]. Our data provide a rationale for the use of these plants in traditional medicine to treat malaria symptoms in Haut-Ogooué province. We are currently attempting to isolate active compounds for testing in animal models of malaria.

## list of abbreviations

CQ: Chloroquine; DHA: Dihydroartemisinin; DELI: Double-site enzyme-linked lactate dehydrogenase immunodetection assay; IC_50_: inhibition concentration 50%, dose which inhibits parasite growth by 50%; CC_50_: **cytotoxic concentration **50%, dose which inhibits cell growth by 50%.

## Competing interests

The authors declare that they have no competing interests.

We declare that there are no competing financial interests regarding this work.

## Authors' contributions

All authors read and approved the final manuscript. JBLD is senior lecturer at USS and CIRMF. He conceived this work, conducted the study and wrote the article. SLOL: was PhD student; she measured antiplasmodial activity. JBB is senior lecturer at USTM: he conceived this work, conducted the chemical part of the study and wrote the article. RZ is a research engineer; she conducted the study and wrote the paper. JL is head of a chemistry research unit; he coordinated the ethnobotanical survey and the chemical part of the study, and wrote the paper. FSTN was head of UPARAM; he coordinated the study and wrote the paper.
